# National age group trends in *Clostridium difficile* infection incidence and health outcomes in United States Community Hospitals

**DOI:** 10.1186/s12879-016-2027-8

**Published:** 2016-11-17

**Authors:** Ashley Pechal, Kevin Lin, Stefan Allen, Kelly Reveles

**Affiliations:** 1 College of Pharmacy, The University of Texas at Austin, 7703 Floyd Curl Drive, MC-6220, San Antonio, TX 78229 USA; 2 Pharmacotherapy Education and Research Center, University of Texas Health Science Center at San Antonio, 7703 Floyd Curl Drive, MC-6220, San Antonio, TX 78229 USA

**Keywords:** *Clostridium difficile* infection, Trends, Incidence, Hospitals, Age group

## Abstract

**Background:**

Prior studies have demonstrated an increase in *Clostridium difficile* infection (CDI) incidence in the United States (U.S.) in recent years, but trends among different age groups have not been evaluated. This study describes national CDI incidence by age group over a 10-year period and mortality and hospital length of stay (LOS) among patients with CDI.

**Methods:**

This was a retrospective analysis of the U.S. National Hospital Discharge Surveys from 2001 to 2010. Eligible patients with an ICD-9-CM discharge diagnosis code for CDI (008.45) were stratified by age: <18 years (pediatrics), 18–64 years (adults), and  ≥65 years (elderly adults). Data weights were used to derive national estimates. CDI incidence was calculated as CDI discharges/1000 total discharges. Mortality and LOS were compared between age groups using chi-square or Wilcoxon rank sum tests.

**Results:**

These data represent 2.3 million hospital discharges for CDI over the study period. CDI incidence was highest among elderly adults (11.6 CDI discharges/1000 total discharges), followed by adults (3.5 CDI discharges/1000 total discharges) and pediatrics (1.2 CDI discharges/1000 total discharges). The elderly also had higher rates of mortality (8.8%) compared to adults (3.1%) and pediatrics (1.4%) (*p* < 0.0001). In addition, median hospital LOS was highest in the elderly (8 days) compared to adults (7 days) and pediatrics (6 days) (*p* < 0.0001).

**Conclusions:**

CDI incidence among patients hospitalized in U.S. hospitals differed based on age group between 2001 and 2010. CDI incidence, mortality, and hospital LOS were highest in the elderly adult population.

## Background

*Clostridium difficile* is the most prevalent pathogen among healthcare-associated infections and recognized by the Centers of Disease Control and Prevention (CDC) as one of the top three urgent threats to public health [1, 2]. *C. difficile* infection (CDI) often presents as diarrhea, but can result in more severe clinical manifestations like toxic megacolon, intestinal perforation, and sepsis. Additionally, CDI can result in death (approximately 29,000 deaths in 2011) [3], prolonged patient hospital stays [4–6], and a marked increase in the economic burden on the healthcare system with mean attributable costs ranging from $8,911 to $30,049 per patient [7].

CDI incidence nearly doubled in United States (U.S.) community hospitals between 2001 and 2010 [8]. A similar report by the Agency for Healthcare Research and Quality (AHRQ) estimated that the number of hospitalizations due to CDI in the U.S. increased almost four times from 1993 to 2009 with the majority of cases affecting individuals 65 and older [9]. Prior studies have affirmed that CDI disproportionately affects the elderly, likely due to immunosuppression from advanced age or chronic comorbidities, more health care exposures, and greater antibiotic use [10, 11]. Despite this, it is unknown if incidence trends differ among age groups [3, 12]. Furthermore, trends in CDI health outcomes have not been explored by age group.

The objectives of this study were to: 1) identify national CDI incidence trends by age group and 2) describe mortality and hospital length of stay (LOS) among CDI patients by age group over a 10-year period.

## Methods

This study utilized data from the CDC’s National Hospital Discharge Surveys (NHDS) from 2001 to 2010. The NHDS survey design and variable definitions have been described previously [13]. Several prior infectious diseases epidemiological studies, specifically evaluating healthcare-associated infections, have utilized NHDS data [8, 14, 15]. The UT Health Science Center San Antonio Institutional Review Board waived formal ethics approval and patient consent, as these data are publically available and do not contain any patient identifiers.

This was a retrospective analysis of patients discharged from U.S. hospitals from 2001 to 2010. Patients were included if they had any (principal or secondary) *International Classification of Diseases, 9* ^*th*^ *Revision, Clinical Modification* (ICD-9-CM) discharge diagnosis code for CDI (008.45). The cohort was categorized into three age groups: pediatrics (<18 years), adults (18–64 years), and elderly adults (≥65 years).

Baseline patient demographics, as provided by the NHDS variable categories, were summarized using median (interquartile range) for continuous variables and counts (percentages) for categorical variables. Annual CDI incidence rates were calculated between 2001 and 2010 using CDI discharges in each age group as the numerator and total discharges in each age group as the denominator. Data weights were applied to derive national estimates. Patient mortality represented all-cause, in-hospital mortality and was identified by the “discharge status” variable of the NHDS. Hospital LOS was identified using the “days of care” variable of the NHDS.

CDI incidence was compared by age group using the z-score. Patient baseline characteristics, mortality, and hospital LOS were compared by age group using appropriate bivariable statistics (chi-square test for categorical variables and Wilcoxon rank sum test for continuous variables). JMP 10.0® (SAS Corp, Cary, NC) was used for all statistical comparisons.

## Results

### Baseline characteristics

Approximately 2.3 million CDI discharges over the study period were included for analysis. Table 1 describes the patients’ baseline demographics and hospital, payment, and admissions characteristics. Of these patients, 67.5% were elderly adults, 28.9% were adults, and 3.6% were pediatrics. The patient population was predominately female (58.7%) and White (85.6%), with a median (IQR) age of 74 (59–83) years. The median age for each group was as follows: pediatrics (6 years), adults (53 years), and elderly adults (80 years). Adult, elderly adult, and pediatric patients with CDI significantly differed with respect to patient sex, race, hospital size and ownership, principal payment source, and admission type and source (*p* < 0.0001 for all) (Table 1). The principal payment source was Medicare for 67% of patient discharges, as expected by the high proportion of elderly adult discharges in the study population. The primary admission source was through the emergency room for adults (60.3%) and elderly adults (59.8%); however; pediatric patients were more likely to be admitted by referral (39.2%).

**Table 1:** Baseline characteristics

Demographic	Overall (*n* = 2,279,004)	Elderly (*n* = 1,538,933)	Adults (*n* = 657,513)	Pediatrics (*n* = 82,558)	*P*-value^*^
Age (years), median (IQR)	74 (59–83)	80 (74-85)	52 (43-59)	5 (2–11)	<0.0001
Female sex	58.7%	61.5%	54.4%	39.4%	<0.0001
Race					
White	85.6%	89.6%	77.2%	78.7%	<0.0001
Black	10.1%	7.0%	16.7%	14.8%	
Other	4.3%	3.4%	6.1%	6.5%	
Hospital size					
6–99 beds	19.4%	22.6%	13.7%	5.6%	<0.0001
100–199 beds	21.1%	21.6%	20.4%	19.1%	
200–299 beds	24.3%	24.8%	21.6%	36.1%	
300–499 beds	22.7%	20.7%	27.1%	25.1%	
500+ beds	12.5%	10.3%	17.2%	14.1%	
Hospital ownership					
Proprietary	11.7%	13.5%	8.2%	5.7%	<0.0001
Government	8.6%	6.8%	12.9%	8.0%	
Nonprofit	79.7%	79.7%	78.9%	86.3%	
Principal payment source					
Medicare	67.0%	88.3%	25.9%	0.0%	<0.0001
Medicaid	8.1%	1.3%	18.7%	50.9%	
Private	21.4%	9.2%	47.1%	43.1%	
Self-pay	1.6%	0.4%	4.6%	0.1%	
Other	1.9%	0.8%	3.7%	5.9%	
Admission type					
Emergency	64.9%	66.4%	64.0%	41.5%	<0.0001
Urgent	21.5%	19.9%	24.1%	33.4%	
Elective	13.4%	13.7%	11.9%	20.6%	
Newborn	0.2%	0.0%	0.0%	4.5%	
Admission source					
Emergency room	59.3%	59.8%	60.3%	37.6%	<0.0001
Transfer	15.1%	18.0%	8.8%	8.6%	
Referral	18.1%	15.7%	21.6%	39.2%	
Other	7.5%	6.5%	9.3%	14.6%	

*IQR* Interquartile range

^*^ *P*-values reflect comparisons between elderly adult, adult, and pediatric CDI populations

### CDI incidence by age group

**Fig. 1:**
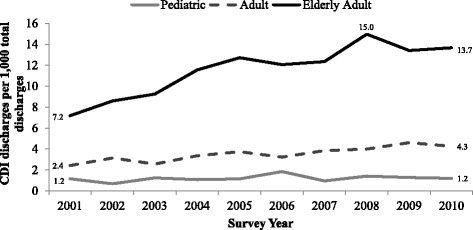
CDI incidence among hospitalized patients by age group in the United States from 2001 to 2010

Overall, CDI incidence significantly differed among age groups (*p* < 0.0001) (Table 2). CDI incidence was higher for elderly adults (11.6 CDI discharges/1000 total discharges), compared to adults (3.5 CDI discharges/1000 total discharges) and pediatric populations (1.2 CDI discharges/1000 total discharges). Elderly adults experienced the greatest change in CDI incidence over the study period (from 7.2 CDI discharges/1000 elderly adult discharges in 2001 to 13.7 CDI discharges/1000 elderly adult discharges in 2010) (Fig. 1). Among adults, CDI incidence increased over the study period, from 2.4 CDI/1000 adult discharges in 2001 to 4.3 CDI discharges/1000 adult discharges in 2010. CDI remained relatively unchanged among pediatrics over the study period (1.2 CDI/1000 pediatric discharges in 2001 and 2010).

**Table 2:** CDI incidence and health outcomes by age group

Outcome	Overall	Elderly	Adults	Pediatrics	*P*-value
CDI cases	2,279,004	1,538,933	657,513	82,558	--
CDI incidence^a^	4.5	11.6	3.5	1.2	<0.0001
Mortality	6.9%	8.8%	3.1%	1.4%	<0.0001
Hospital LOS	8 (4–14)	8 (5–14)	7 (4–14)	6 (3–16)	<0.0001

^a^CDI incidence calculated at CDI discharges per 1000 total discharges

### Mortality and hospital LOS among patients with CDI by age group

The overall all-cause, in-hospital mortality was 6.9% and the median (IQR) hospital LOS was 8 (5–14) days. Mortality and hospital LOS significantly differed by age group (*p* < 0.0001 for each comparison). Mortality was higher for elderly adults (8.8%) as compared to adult (6.9%) and pediatric populations (3.1%) (*p* < 0.0001). Among those patients with CDI who died, the most common co-mortalities were septicemia (ICD-9-CM code 038.X; 35.7%), acute renal failure (ICD-9-CM code 584.X; 31.8%), pneumonia (ICD-9-CM codes 480–486.XX; 22.3%), and urinary tract infection (ICD-9-CM codes 599.0; 18.8%). Similar to mortality, median hospital LOS was longest for elderly adults (8 days), followed by adults (7 days), and pediatrics (6 days) (*p* < 0.0001). There were no differences between male and female elderly CDI patients for mortality (8.8% for both sexes) or median hospital LOS (8 days for both sexes).

## Discussion

Prior national epidemiological investigations demonstrated considerable increases in CDI incidence over the last decade [3, 15, 16]; however, this is the first study to document the national burden of CDI longitudinally among different age groups. This report identified a disproportionate rise in CDI incidence among elderly adults, with an estimated 61% rate of increase from 2001 to 2010. Notably, elderly adults also suffered significantly higher all-cause, in-hospital mortality and increased hospital LOS compared to adult and pediatric patients.

Several factors could contribute to the disparate CDI incidence increase and health outcomes among the elderly compared to other age groups. Advanced patient age has been previously linked to an increase risk for CDI development. This is likely attributed to innate and iatrogenic changes such as: immunosenescence [17], higher prevalence of comorbid illness [18], changes in the gut flora [17], more healthcare exposures (e.g*.*, hospitalizations and long-term care facility residence), and exposure to antibiotics [19] and other medications (e.g*.*, proton pump inhibitors [PPIs]) [20]. In recent years, there has been an increase in the use of certain antibiotics, particularly among the elderly. Lee et al. [21] demonstrated an increase in overall antibiotic use in older adult patients by 30% and use of broad spectrum antibiotics in elderly adults by 68% from 2000 to 2010. Furthermore, two meta-analyses suggest that PPI use is associated with increased risk for CDI [22, 23]. Often, patients take PPIs inappropriately [24, 25]. Choudhry et al. [25] found that in a predominately elderly population (median age 76 years), more than half (53.4%) were prescribed a PPI without an appropriate indication. Furthermore, 7.9% were prescribed a PPI for unknown reasons. In addition, there has been a dramatic increase in the use of PPIs among outpatients in the U.S. A 2013 study by Rotman et al. [24] found that the use of PPIs more than doubled among outpatients in the U.S. between 2002 and 2009.

The poorer health outcomes among elderly patients with CDI could be due to several factors. First, the European and North American CDI guidelines report age over 65 years as a marker of severity [26, 27]. Severity of infection has been previously linked with increased patient mortality, as well as longer hospital LOS [28]. Additionally, a prior study demonstrated an increased risk of severe infection and death due to the more pathogenic *C. difficile* strain, BI/NAP1/027 strain in elderly adults compared to younger populations [29].

Our study findings are important for several reasons. First, in 2001, elderly adults represented approximately 13% of the U.S. population. During our study period, there was an addition of approximately five million elderly adults to the U.S. population [30]. By the year 2030, it is expected that elderly adults will grow to 19% of the total U.S. population. As the population ages, a greater proportion of Americans become high-risk for developing CDI. The incidence and health outcome trends elucidated in our study may help increase awareness of CDI, identify and protect high risk patients, and possibly reduce the occurrence of CDI in the hospital setting.

## Conclusions

CDI incidence was highest among elderly adults at least 65 years of age, followed by adults and pediatric patients. The increase in CDI incidence among the elderly markedly outpaced that of the other two age group populations from 2001 to 2010. Additionally, elderly adults experienced higher all-cause, in-hospital mortality and longer hospitals stays as compared to adults and pediatrics patients.

### Limitations

This study has potential limitations. First, this study was retrospective and relied on administrative coding to identify CDI cases rather than positive laboratory identification of *C. difficile*, which could result in misclassification bias. Although the use of ICD-9-CM codes to identify CDI has relatively high specificity (99.7%) and sensitivity (78%), it cannot be considered equivalent to medical chart review or microbiological analysis [31]. Importantly, our CDI case definition did not change over the study period; therefore, any coding error would persist throughout the study years and would have limited effects on CDI trends. Additionally, an initial CDI episode could not be discriminated from a recurrent CDI episode or readmission. Furthermore, there are factors that could have contributed to differences in CDI incidence and outcomes between age groups that we were not able to account for in our analyses including: antibiotic exposure, differences in CDI testing procedures, severity of comorbid illness, and *C. difficile* strain/ribotype. The exclusion of federal hospitals and long-term care hospitals in the NHDS and the lack of data after 2010 potentially limit the generalizability to those settings and may underestimate the burden of CDI in the U.S., particularly in recent years. Finally, the NHDS includes a large sample size, resulting in high study power. This limits the utility of *p*-values to establish differences among groups, as small variations are likely to be statistically significant.

## Abbreviations

AHRQ: Agency for Healthcare Research and Quality
CDC: Centers for Disease Control and Prevention
CDI: *Clostridium difficile* infection
LOS: Length of stay
NHDS: National Hospital Discharge Survey
PPI: Proton pump inhibitor
